# Connexin 50 Functions as an Adhesive Molecule and Promotes Lens Cell Differentiation

**DOI:** 10.1038/s41598-017-05647-9

**Published:** 2017-07-13

**Authors:** Zhengping Hu, Wen Shi, Manuel A. Riquelme, Qian Shi, Sondip Biswas, Woo-Kuen Lo, Thomas W. White, Sumin Gu, Jean X. Jiang

**Affiliations:** 1 Departments of Biochemistry and Structural Biology, University of Texas Health Science Center, San Antonio, TX USA; 2 The Second Xiangya Hospital, Central South University, Changsha, China; 3 Departments of Physiology, University of Texas Health Science Center, San Antonio, TX USA; 4 Department of Neurobiology, Morehouse School of Medicine, Atlanta, GA USA; 5 Department of Physiology and Biophysics, Stony Brook University, Stony Brook, NY USA

## Abstract

Connexins play essential roles in lens homeostasis and development. Here, we identified a new role for Cx50 that mediates cell-cell adhesion function. Cx50 enhanced the adhesive capability of AQP0. Interestingly, the expression of Cx50 alone promoted cell adhesion at a comparable level to AQP0; however, this cell adhesive function was not observed with other lens connexins, Cx43 and Cx46. Moreover, the adhesive property occurred in both homotypic with Cx50 expressed in both pairing cells and heterotypic with Cx50 in only one pairing cell, and this function appears to be unrelated to its role in forming gap junction channels. Cx50 KO lenses exhibited increased intercellular spaces between lens fiber cells. The second extracellular loop domain (E2) is primarily responsible for this adhesive function. Treatment with a fusion protein containing E2 domain inhibited cell adhesion. Furthermore, disruption of cell adhesion by the E2 domains impaired primary lens cell differentiation. Five critical amino acid residues in the E2 domain primarily are involved in cell adhesive function as well as lens epithelial-fiber differentiation. Together, these results suggest that in addition to forming gap junction channels, Cx50 acts as an adhesive molecule that is critical in maintaining lens fiber integrity and epithelial-fiber differentiation.

## Introduction

Gap junctions that connect the cytoplasm of adjacent cells and permit passage of metabolites, ions and second messengers play essential roles in lens homeostasis and transparency. Gap junctions are formed by a family of membrane proteins called connexins^1^, which have four conserved transmembrane and two extracellular loop (E) domains and, a variable intracellular loop (IL) and a C-terminal (CT) domains. Three major connexins have been identified in the vertebrate lens; Cx43, Cx46 and Cx50. Mutations of Cx46 and Cx50 genes are the most common causes of congenital cataracts in humans. Similar lens phenotypes ware reported in connexin-deficient or mutation murine models^2, 3^. Our previous studies have shown that Cx50, but not Cx46 or Cx43, associates with aquaporin 0 (AQP0), the most abundant membrane protein in the differentiating, but not mature lens fibers^4^. This interaction promotes gap junctional channel activity^5^, and the IL domain of Cx50 and the CT domain of AQP0 directly interact with each other^6^.

The lens is an avascular organ, which is formed by an anterior epithelial cell layer and highly differentiated fiber cells. Epithelial cells located at the lens equator differentiate to lens fiber cells, which gradually lose their intracellular nuclei and organelles in lens development. During this process, mature lens fibers accumulate high concentrations of AQP0, crystallins, Cx46 and Cx50. Because of the lack of vasculature, the lens is dependent upon an extensive network of gap junction intercellular communication to maintain lens homeostasis^7^. AQP0, also known as major intrinsic protein (MIP), is the most abundant membrane protein expressed in lens fibers. However, unlike other members of aquaporin family, water permeability of mammalian AQP0 is remarkably low, estimated to be 40-times lower than that of the AQP1 channel in lens anterior epithelial cells^8^, while zebrafish AQP0 has high water permeability similar to mammalian AQP1^9^. Besides functioning as a water channel, AQP0 plays a crucial structural role as an adhesion molecule in mediating the formation of thin junctions between lens fibers^10–13^. In addition, AQP0 interacts with several proteins, such as calmodulin^14^, intermediate filament proteins filensin and CP49^15^, as well as γ-crystallins^16, 17^. Although connexin molecules have been implied to be involved in facilitating cell-cell interaction due to their formation of gap junctions between adjacent cells, there is a scarcity of knowledge with regards to the direct cell adhesive function of connexins.

In this study, we show that Cx50, unlike two other lens connexins, Cx43 and Cx46, mediates cell adhesion function through its second extracellular loop domain. Moreover, the cell-cell adhesion mediated by Cx50 plays a critical role for lens epithelial-fiber cell differentiation.

## Results

### Cx50 Exhibits Cell-cell Adhesion Function and Enhances the Adhesive Capability of AQP0

**Figure 1:**
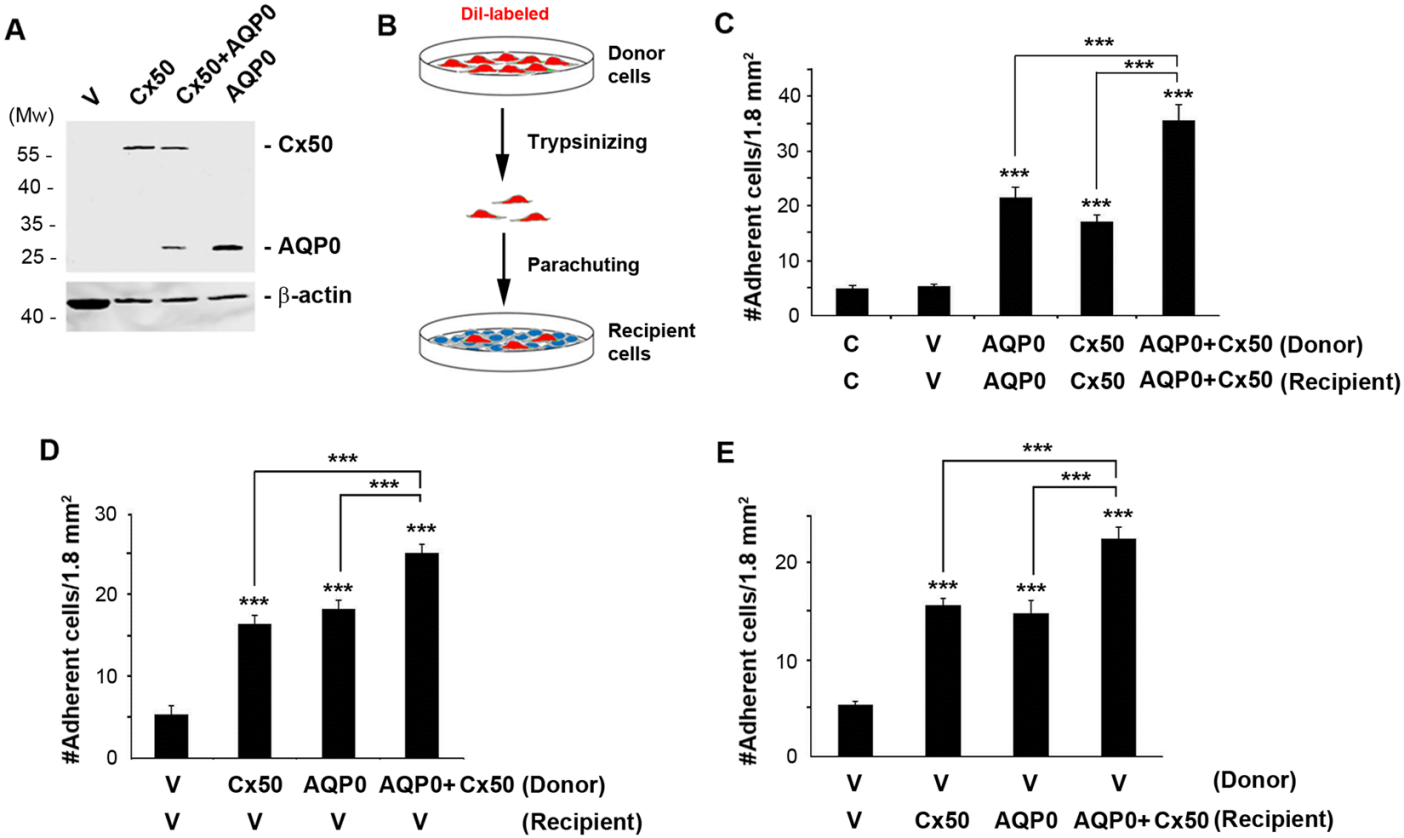
Cx50 increases cell-cell adhesion and enhances the adhesive capability of AQP0. (**A**) Crude membrane extracts were prepared from CEF cells infected with high titer RCAS(A) retroviral vehicle (V) or recombinant RCAS(A) retroviruses containing Cx50, AQP0 or co-infected with both Cx50 and AQP0 and were immunoblotted with anti-Flag or β-actin antibody. (**B**) The diagram illustrates cell adhesion assay via “parachuting” Dil-fluorescence-labeled donor cells onto the confluent recipient cells. (**C–E**) High titer recombinant retroviruses containing RCAS(A) vehicle (V), Cx50 or AQP0 were used to infect CEF cells. Cell-cell adhesion assay with donor and receipt cells infected with various combinations of recombinant retroviruses containing Cx50 or/and AQP0. After 1.5 hr incubation, fluorescent adherent donor cells were counted and quantified. The data are presented as the mean ± SEM. n = 3. As compared to vehicle controls (V), ***P < 0.001.

We have shown that Cx50 interaction with AQP0 enhances gap junctional coupling^5, 6^. To explore if Cx50 has any effect on the cell adhesion function of AQP0, we conducted a cell adhesion assay using chicken embryonic fibroblast (CEF) cells, a cell line deficient in lens connexins and AQP0^18^, and cannot form functional gap junction channels between themselves and between parental CEF and the CEF expressing exogenous Cx50 (Fig. S1). Exogenous Cx50 and AQP0 were expressed in CEF cells via retroviral infection (Fig. 1A). The cell adhesion assay was then performed by “parachuting” Dil-labeled donor cells to the confluent recipient cells as illustrated in Fig. 1B. We expressed Cx50 and/or AQP0 in various combinations in donor and receipt cells. As compared to CEF cells only (C) and RCAS(A) vehicle (V) controls, the presence of AQP0 significantly increased the number of adherent cells when it was expressed in both donor and recipient cells (homotypic) (Fig. 1C) as well as when it was only present in either recipient or donor cells (heterotypic) (Fig. 1D and E). Similarly, co-expression of AQP0 with Cx50 further enhanced the numbers of adherent cells when expressed in a heterotypic or homotypic manner (Fig. 1C–E). Surprisingly, we observed that Cx50, by itself, significantly increased cell adhesion, and also acted in either a homotypic or heterotypic manner (Fig. 1C–E). There is no statistical difference when comparing the cell adhesion by homotypic AQP0 and Cx50. These results suggest that co-expression of Cx50 further enhances the adhesive function of AQP0 and more importantly, Cx50 by itself possess cell adhesion function similar to AQP0.

**Figure 2:**
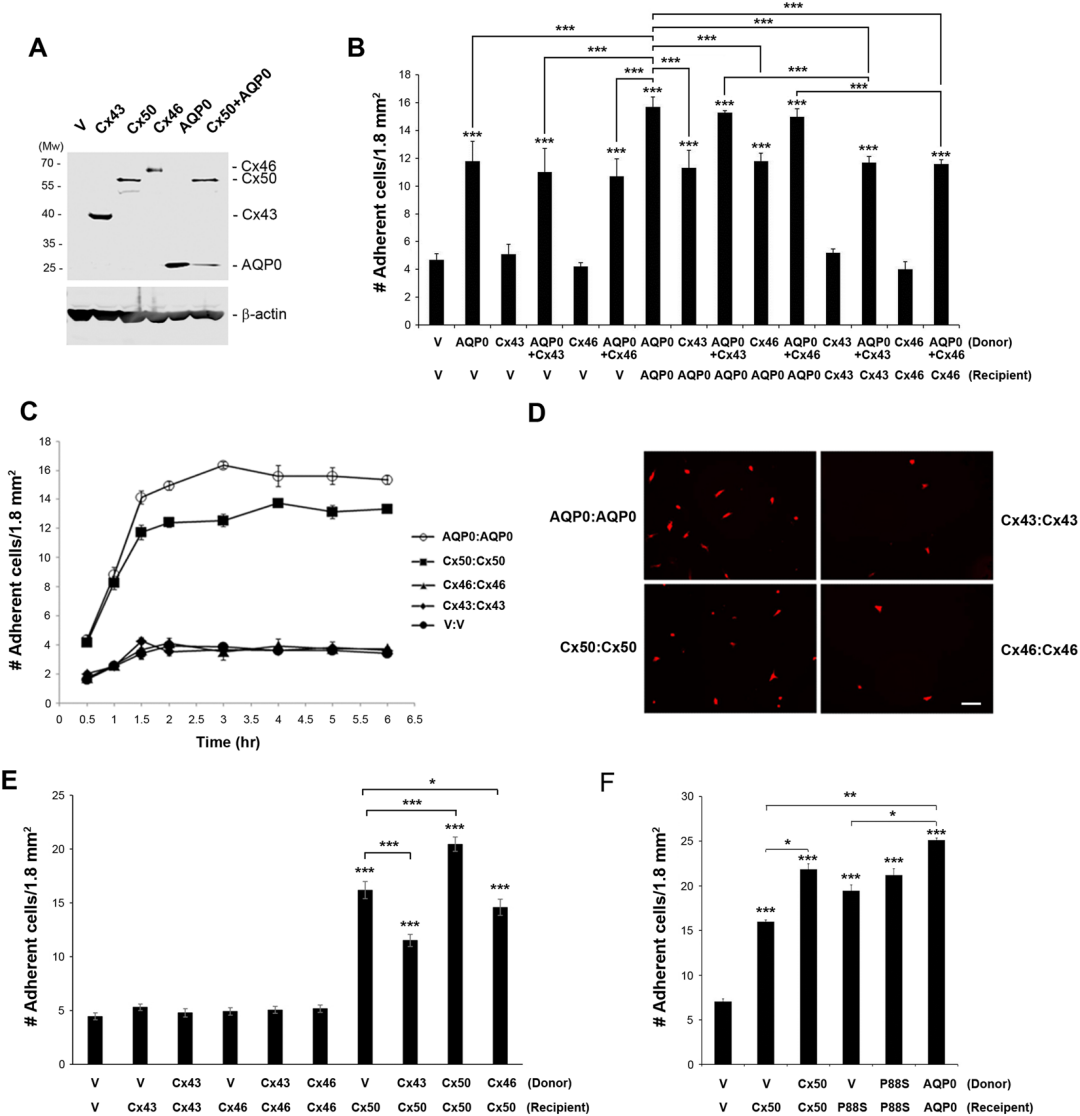
Cx50, not the other two lens connexins, Cx43 and Cx46, mediates cell-cell adhesion, and cell adhesion function is independent of Cx50 channel-forming function. Retroviruses containing RCAS(A) vehicle (V), Cx50, Cx46, Cx43 or AQP0 were used to infect CEF cells. (**A**) Crude membrane extracts of CEF cells expressing various connexins or/and AQP0 were immunoblotted with anti-Flag or β-actin antibody. (**B**) Cell-cell adhesion assay was conducted with donor or recipient cells expressing various combinations of connexins and AQP0. After 1.5 hr incubation, adherent cells were counted and quantified. (**C**) Cell adhesion assay was performed in CEF cells homotypic expressing AQP0, Cx50, Cx43, Cx46 or vehicle (V) control after various time periods of incubation. Adherent cells were counted and quantified. (**D**) Representative fluorescence images of adherent cells are shown in red fluorescence. Bar, 50 µm. (**E**) Cell adhesion assay was performed in CEF cells expressing lens connexins or vehicles (V) homotypic or heterotypic. (**F**) High titter recombinant retroviruses containing RCAS(A) vehicle (V), Cx50, Cx50P88S mutant or AQP0 were used to infect CEF cells. Cell adhesion assay was conducted and adherent cells were counted and quantified. The data are presented as the mean ± SEM. n = 3. As compared to vehicle (V) controls or otherwise indicated, *P < 0.05; **P < 0.01; ***P < 0.001.

To determine if this cell adhesion property is unique to Cx50, we examined two other lens connexins, Cx43 and Cx46. Western blots showed that all three exogenous connexins or in combinations were expressed in CEF cells following retroviral transfection (Fig. 2A) and co-expression reduced the level of the ones expressed alone as we have previously observed^18–20^. However, unlike Cx50, Cx43 and Cx46 did not function as adhesive molecules in either a homotypic or heterotypic manner (Fig. 2B). Moreover, co-expression of Cx43 or Cx46 with AQP0 failed to further enhance the the adhesive function of AQP0. Given that these connexins also form gap junctions between two adjacent cells, the cell adhesion function of Cx50 could be a result of the formation of gap junctions. The lack of cell adhesion function by Cx43 or Cx46 could be due to insufficient time necessary to form gap junctions. To explore this possibility, we conducted cell adhesion assay by counting Dil-labeled adherent cells “parachuting” on recipient cells at various time periods up to 6 hrs. There was a significant increase in cell adhesion within the first 1.5 hrs in cells expressing AQP0 or Cx50, whereas there was a minimal effect in Cx43 or Cx46-expressing cells that was similar to the RCAS(A) vehicle (V) control (Fig. 2C). The difference was clearly shown in representative images of Dil-labeled fluorescent, adherent cells after 1.5 hrs of incubation (Fig. 2D). To further confirm that the adhesive function is solely due to Cx50 and not the other lens connexins, we also performed the cell-to-cell adhesion assay through the heterotypic pairing of connexins (Fig. 2E). The result showed that only cells expressing Cx50, regardless of its homotypic or heterotypic expression, exhibited increased cell adhesion. Intriguingly, more adherent cells were observed in cells expressing heterotypic Cx50 paired with RCAS(A) vehicle (V) control cells than those paired with Cx43 or Cx46-expressing cells. These data indicate that expression of Cx43 or Cx46 on the cell membrane may interfere with Cx50 heterotypic adhesion to other, non-connexin partner(s) on the adjacent cell membrane.

The above evidence indicates that the cell adhesion function of Cx50 appears to be unrelated to its role in forming functional gap junctions, given that both Cx43 and Cx46 also form functional gap junction channels in CEF cells^21^. We further explored this assertion using a Cx50 mutant, P88S, that expresses on the surface of differentiated lens fibers and impairs the ability of Cx50 to form functional gap junction channels and hemichannels^18, 22^. We also showed cell surface localization of this mutant in CEF cells (Fig. S2). However, like WT Cx50, the mutant Cx50P88S had comparable cell adhesive capability and also functioned in both a homotypic and heterotypic adhesion manner (Fig. 2F). Together, these results suggest that only Cx50, and not the other lens connexins, acts as a cell adhesive molecule, which has a role different from forming gap junction channels.

### The Second Extracellular Loop Domain (E2) Is Involved in the Adhesion Function of Cx50

**Figure 3:**
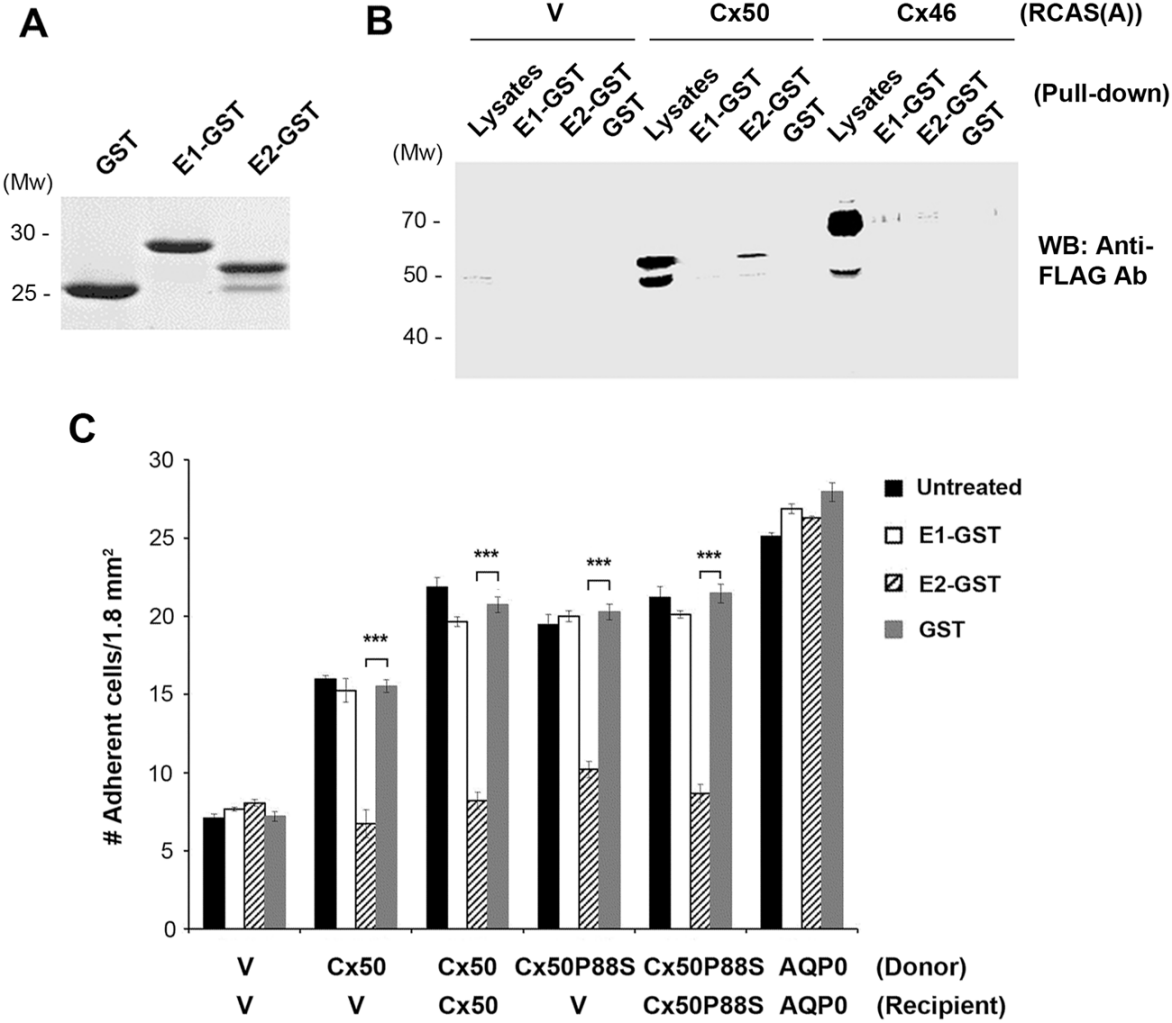
Second extracellular loop domain (E2) Cx50 mediates the adhesion function of Cx50 and this function is independent of forming functional connexin channels. (**A**) GST-fusion protein containing E1 and E2 domains were generated and detected by Commassie blue staining. (**B**) High titter recombinant retroviruses containing RCAS(A) vehicle (V), Cx50 and Cx46 were used to infect CEF cells and cell lysates were prepared. Protein pull down assay was performed using cell lysates and E1-GST, E2-GST or GST attached to glutathione beads. Cell lysates (Lysates) and elutes (E1-GST, E2-GST or GST) from the beads were immunoblotted with anti-FLAG antibody. (**C**) High titter recombinant retroviruses containing RCAS(A) vehicle (V), Cx50, Cx50P88S mutant or AQP0 were used to infect CEF cells in the absence or presence of GST-fusion protein containing E1 or E2 domain. Cell adhesion assay was conducted and adherent cells were counted and quantified. The data are presented as the mean ± SEM. n = 3. ***P < 0.001.

Connexins have two extracellular loop domains, E1 and E2. The sequences of Cx50 E1 and E2 domains, and their sequence comparisons with E1 and E2 domains of Cx43, Cx46 and Cx26, respectively are illustrated (Table 1). To determine which domain(s) mediates cell adhesion function, we generated GST fusion protein containing the E1 or E2 domain of Cx50, or GST as a control (Fig. 3A), and tested its interaction with connexins and effect on cell adhesion mediated by Cx50. Protein pull-down assay using E1, E2 GST-fusion protein or GST as a control showed that E2, not E1 fusion protein or GST, interacted with Cx50 and this interaction was not detected in cell lysates containing Cx46 (Fig. 3B *)*.

**Table 1:** Comparison of chicken Cx50E1 and E2 domains with chicken Cx43, Cx46 and Cx26.

Chicken Cx50 **E1**	45 WGDEQSDFVCNTQQPGCENVCYDEAFPISH 74
Chicken Cx46 **E1**	45 WGDEQSDFTCNTQQPGCENVCYDKAFPISH 74
Chicken Cx43 **E1**	45 WGDEQSAFRCNTQQPGCENVCYDKSFPISH 74
Chicken Cx26 **E1**	44 WGDEQDDFICNTLQPGCKNVCYDHFFPISH 73
Chicken Cx50 **E2**	176 GFRILPLYRCGRWPCPNLVDCFVSRPTEKT 205
Chicken Cx46 **E2**	194 GFELKPVYQCSRPPCPHTVDCFISRPTEKT 223
Chicken Cx43 **E2**	178 GFSLSAIYTCERDPCPHRVDCFLSRPTEKT 207
Chicken Cx26 **E2**	160 GFRMPRLMKCSAWPCPNTVDCFVSRPTEKT 189

CEF cells expressing homotypic or heterotypic Cx50 were pre-incubated with the aforementioned fusion proteins or GST control and the cell adhesion assay was performed. We determined the half-lives of Cx50 E1 and E2-GST proteins in CEF cells expressing Cx50 and found these two fusion proteins are stable in cell culture with half-lives of 30.6 ± 4.5 and 36.4 ± 3.5 hrs, respectively (Fig. S4). The fusion proteins were added to the cell every day. Addition of the GST fusion protein containing E2 (E2-GST) significantly attenuated the enhancement of cell adhesion by Cx50, while the E1-GST and GST had no such effect (Fig. 3C). We also observed a similar inhibitory effect on cell adhesion in cells expressing Cx50P88S mutant. However, this effect was not shown in cells expressing AQP0, suggesting that the inhibition of cell adhesion by E2-GST was specific to Cx50.

**Figure 4:**
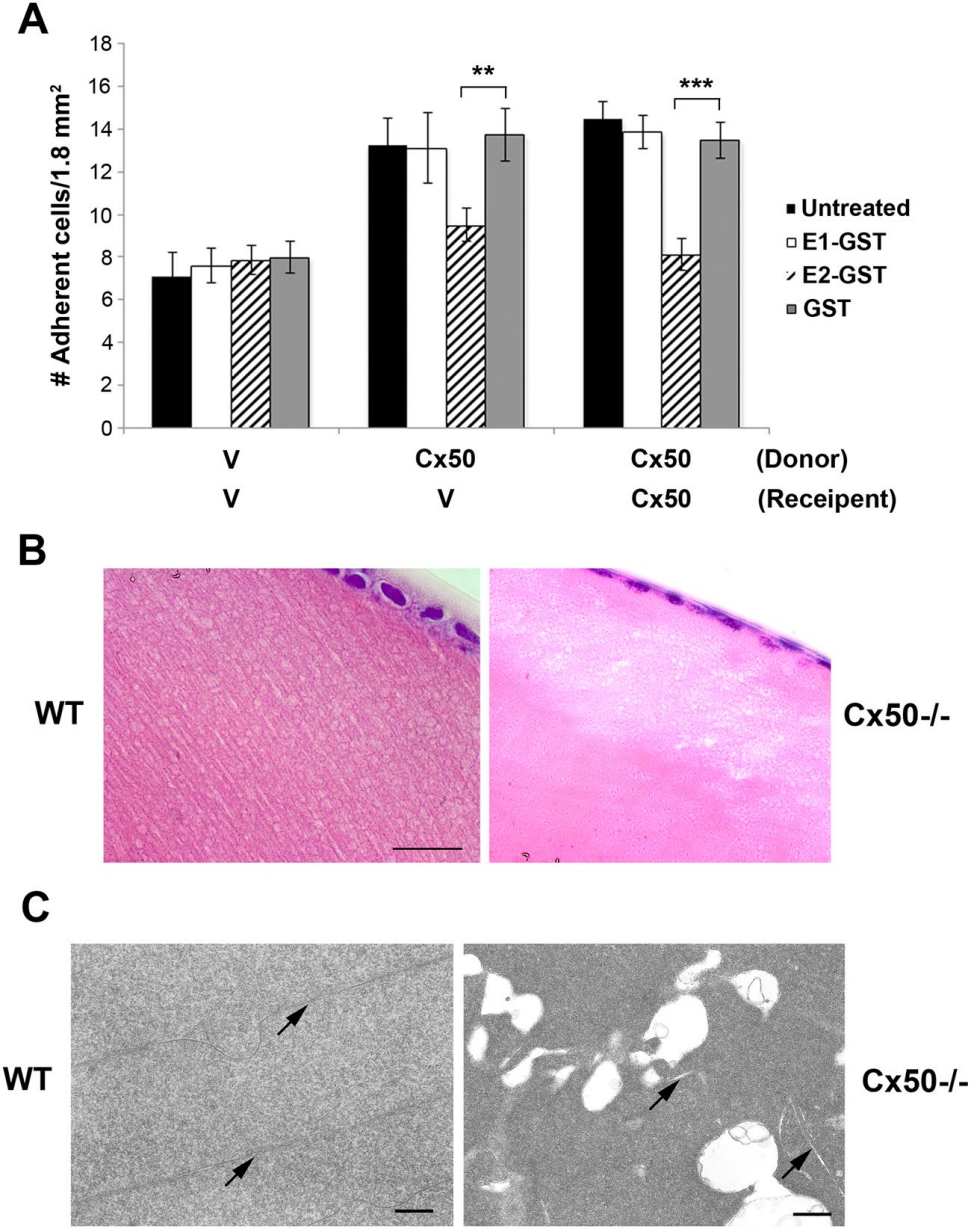
Cx50 mediates cell adhesion function and Cx50 deficiency in lens fiber cells resulted in increased intercellular spaces. (**A**) High titter recombinant lentivirus containing vehicle (V) or Cx50 were used to infect HEK293 cells. Cell adhesion assay was conducted and adherent cells were counted and quantified. The data are presented as the mean ± SEM. n = 3. **P < 0.01; ***P < 0.001. (**B**) H&E staining of paraffin tissue sections of postnatal day 15 mouse lenses of WT or Cx50 KO mice were prepared and representative images were taken from the anterior cortex of the lenses. Bar, 20 µm. n ≥ 3. (**C**) One month old mouse lenses of WT or Cx50 gene KO mice were processed for thin section TEM. Representative images from the anterior superficial cortex of the lenses show that cortical fiber cells exhibit the intact cell membranes (arrows) in WT, but display enlarged intercellular spaces of various sizes in single KO lenses for Cx50. Several short intact cell membranes (arrows) were also observed among these enlarged spaces. All scale bars, 500 nm. n ≥ 3.

We further confirm the adhesive role of Cx50 in other cell types. The mouse ortholog of Cx50 was expressed in HEK193 cells via lentiviral infection and cell adhesion assay was similarly performed. The increased adhesive function by Cx50 was similarly observed in both heterotypic and homotypic manner (Fig. 4A). Given that extracellular loop domains of Cx50 are highly homologous between chicken and mouse, application of the same E1 or E2-GST fusion proteins showed the similar inhibition by E2, but not E1-GST or GST. Together, these data suggest that E2 domain plays an important role in mediating cell adhesion function of Cx50.

We examined the microscopic organization of fiber cells in Cx50 knockout mice. H&E staining of postnatal day-15 mouse lenses showed that compared to WT lenses, Cx50 KO lenses showed disorganized lens fiber structures with some empty spaces, particularly close to the anterior (Fig. 4B) and posterior regions of the lens. Thin section electron microscopy results revealed the presence of numerous vacuole structures between the lens fibers and increased intercellular spaces (arrows) in Cx50 knockout lenses (Fig. 4C). Consistently, a recent paper also reports multiple morphological defects in the cortical fibers of Cx50 knockout lenses^23^. The increase of extracellular spaces is also reported in the lens fibers lacking AQP0^24^. Together, these data support an important role of Cx50 in cell adhesion, which likely leads to the integrity and compact organization of lens fibers.

### Cell Adhesion by Cx50 Plays an Important Role in Lens Epithelial-Fiber Cell Differentiation

**Figure 5:**
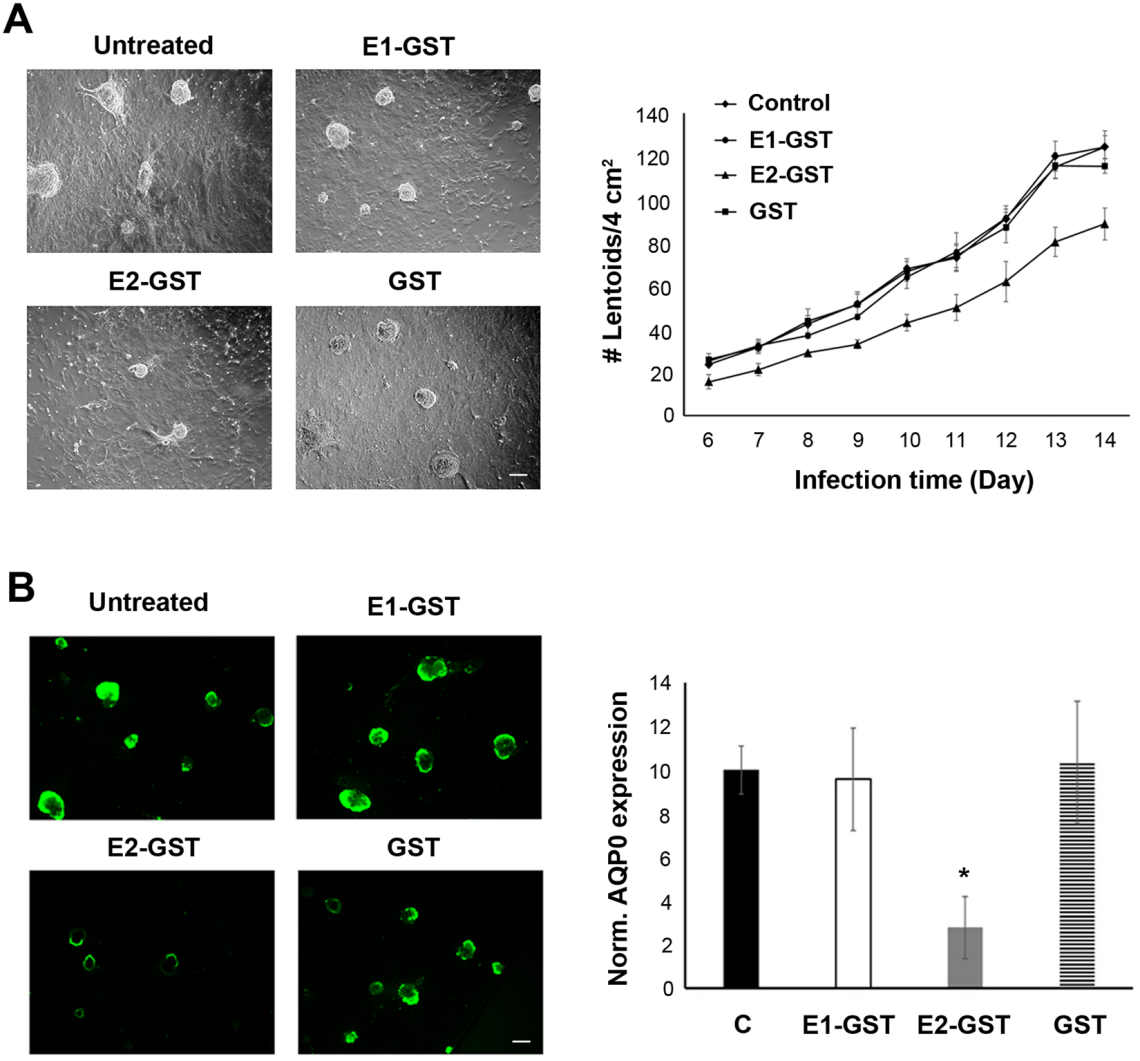
Disruption of cell adhesion by Cx50 E2 domain impairs lens epithelial-fiber cell differentiation. (**A**) Cx50 E1-GST, E2-GST or GST was used to treat primary lens cell culture every day starting at the 2nd day of cell seeding. Representative images of primary culture cells treated with GST-E1, E2 fusion proteins or GST protein and untreated control were taken on the 10^th^ day of primary culturing. Starting at the 6th day of the treatment, the total lentoid numbers were quantified each day until day 14. (**B**) Fourteen days after injection, fixed primary lens cells were immunostained with anti-AQP0 antibody and detected by fluorescein-conjugated anti-rabbit IgG. The signals of AQP0 expression areas versus whole-image areas were quantified. The data are presented as the mean ± SEM. *P < 0.05; **P < 0.01; ***P < 0.001. n = 3. Bar, 200 µm.

Cx50 is directly involved in lens epithelial to fiber cell differentiation as well as lens development based on both *in vivo* KO models and *in vitro* studies with primary lens cells^25–27^. Here, we explored if the cell adhesion function of Cx50 is crucial to lens fiber differentiation. Cultured chick lens primary cells undergo morphological changes associated with the formation of lentoid structures and the expression of lens differentiation marker proteins, a process closely mimicking lens epithelial to fiber cell differentiation *in situ* ^28^. We treated these primary cultures with a GST fusion protein containing either the E1 or E2 domain of Cx50 or GST as control starting at the 3–4^th^ day after cell seeding. Lentoids started to emerge around day 6 and continued until day 14. Representative images of lentoids were taken (Fig. 5A, left panels) and the number of lentoids was counted each day (Fig. 5A, right panel). The lentoid numbers were reduced in cultures treated with E2-GST while the untreated control, E1-GST- or GST-treated cultures had similar, time-dependent increases in lentoid numbers. To further quantify the extent of lens cell differentiation, we examined the expression level of AQP0, a marker for lens cell differentiation and maturation, at day 14 after primary cell culturing. Immunofluorescence images showed a reduction of AQP0 expression only in E2-GST treated cells, but not in those treated with E1-GST, GST or untreated control (Fig. 5B, left panels). Quantification of fluorescence intensity further confirmed the decrease of AQP0 expression (Fig. 5B, right panel). These data suggest that Cx50-mediated cell adhesion plays a critical role in lens epithelial to fiber cell differentiation.

### Identification of Amino Acid Residues at E2 Domain in Cell Adhesive and Lens Cell Differentiation Function of Cx50

**Figure 6:**
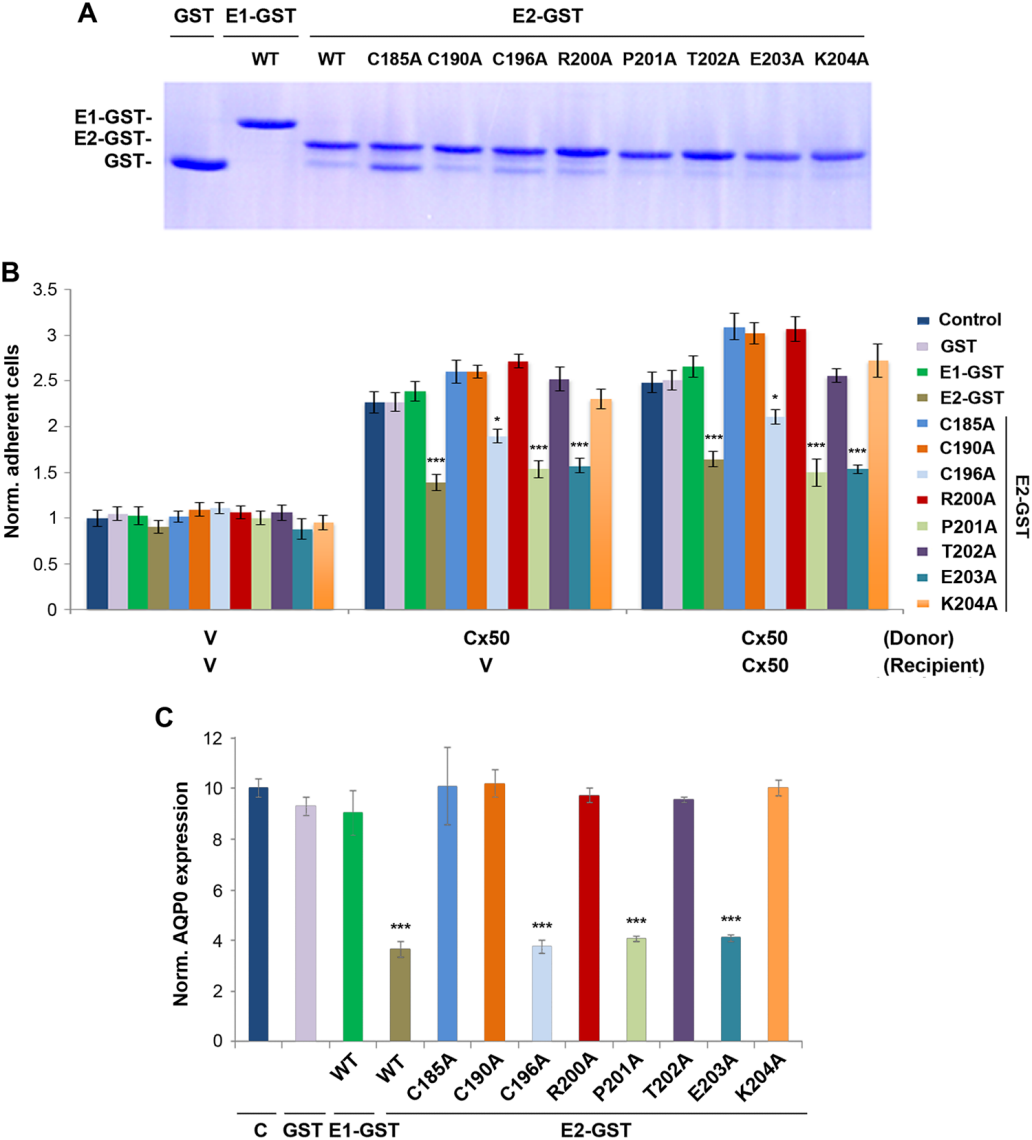
Identification of critical amino acid residues at E2 domain involved in cell adhesion and lens epithelial-fiber differentiation. (**A**) GST-fusion proteins containing WT E2, and various single site mutation of E2 domain were generated and detected by Commassie blue staining. (**B**) High titter recombinant retroviruses containing RCAS(A) vehicle (V) and Cx50, were used to infect CEF cells in the absence or presence of GST-fusion protein containing WT or 8 single site-mutated E2 domain. Cell adhesion assay was conducted and adherent cells were counted and quantified. The data are presented as the mean ± SEM. n = 3. As compared to untreated controls, *P < 0.05; ***P < 0.001. (**C**) GST fusion proteins containing WT or single site mutated E2 domain or GST was used to treat primary lens cell culture every day starting at the 2nd day of cell seeding. Fourteen days after injection, fixed primary lens cells were immunostained with anti-AQP0 antibody and detected by fluorescein-conjugated anti-rabbit IgG. The signals of AQP0 expression areas versus whole-image areas were quantified. The data are presented as the mean ± SEM. n = 3. As compared to untreated control (**C**), ***P < 0.001.

To determine the critical amino acid residues that are involved in cell adhesion and lens fiber differentiation, we mutated 8 conserved amino acid residues located in E2 domains; 3 cysteine conserved residues important for the formation of intramolecular disulfide bonds between E1 and E2 domains, and 5 residues which are conserved in Cx50 across species by site-directed mutagenesis. We generated GST fusion proteins with mutation of these residues to alanine. Cell adhesion assay was performed in Cx50-expressing CEF cells in the absence or presence of GST fusion proteins containing WT or mutant E2 domains. The comparable levels of E2-GST fusion proteins containing various single site mutation or WT (Fig. 6A) were incubated with CEF cells expressing Cx50 in heterotypic or homotypic manner or RCAS(A) vehicle (V) control. Mutant fusion proteins C196A, P201A and E203A had significant inhibition on cell adhesion as that containing WT E2 domain, while C185A, C190A, R200A, T202A and K204A had no such effects (Fig. 6B and summarized in Table 2). Additionally, we found a similar level of the inhibition of Cx50 adhesion by Cx43E2 fusion protein that also contains these five conserved residues (Fig. S5). The E2 fusion proteins containing these site mutations were applied to lens primary culture and the extent of lens cell differentiation was analyzed. Consistent with the result of cell adhesion, the identical 5 mutants of E2 domain failed to attenuate the inhibitory effect of E2-GST on lens fiber differentiation, indicated by the expression of AQP0 (Fig. 6C). These data support the notion that cell adhesion function mediated by critical amino acid residues of E2 domain is indispensable for lens fiber cell differentiation.

**Table 2:** Summary of the effect of mutants.

Cx50 E2 mutations	C185A	C190A	C196A	R200A	P201A	T202A	E203A	K204A
Blocking Adhesion	−	−	+	−	+	−	+	−
Blocking Differentiation	−	−	+	−	+	−	+	−

## Discussion

Integrity and precise organization of lens fiber cell structures is crucial for light transmission and optical quality, and optimal cell adhesion between lens fiber cells is an essential part of this process^29^. We previously reported the role of AQP0 in promoting gap junction coupling mediated by Cx50^5^. However, the potential involvement of Cx50 in the role of AQP0 in cell adhesion function remains largely elusive. Our cell-to-cell adhesion assay showed that co-expression of AQP0 and Cx50 further enhanced the adhesive function of AQP0. Interestingly, Cx50 by itself acts as an adhesive molecule and its adhesive capacity is comparable to that of AQP0. This is the first time that Cx50 has been shown to function as an adhesive molecule besides its role in forming connexin channels. The enlarged intercellular spaces between fiber cells observed in the lens of Cx50 KO mice are likely to be a consequence of deficient adhesive function of Cx50.

Interestingly, this adhesive function acts in both homotypic (with both pairing cells expressing Cx50) and heterotypic (either donor or recipient cell expressing Cx50) fashion. These data indicate that gap junction formation requiring connexin expression in both paired cells is unlikely to be involved in the process of cell adhesion. To test this possibility and the specificity of cell adhesion by Cx50, we used Cx43 and Cx46, the other two connexins present in the lens. Unlike Cx50, these two connexins failed to exhibit any cell adhesive function, given that Cx43 and Cx46 form gap junctions and mediate cell-cell coupling^5^. We then conducted dynamic cell-cell adhesion studies to explore the possibility that the formation of gap junctions between two adjacent cells by the other lens connexins may take a longer period of time than Cx50. However, the data showed a time-dependent increase in cell adhesion only in Cx50 and not the other lens connexin-expressing cells, further eliminating the involvement of gap junction formation in cell-cell adhesion.

The cell adhesive function of connexins has been proposed over the decades, particularly because they have a close association with other cell adhesion proteins including tight junction proteins, cadherins and other cytoskeletal proteins^30^. For example, gap junctions are suggested to function analogously to cell adhesion molecules in mediating cellular recognition and selective neurite adhesion and repulsion^31^. However, these reported adhesive properties are primarily dependent upon the establishment of gap junction structures with the expression of the same connexin in both paired cells^32^. We further excluded the involvement of functional gap junction coupling since the Cx50P88S mutant that fails to mediate cell coupling possesses the same adhesive capability as WT Cx50. This study also diminishes the possible involvement of functional hemichannels since Cx50P88S mutant also inhibits this type of connexin channel in a dominant negative manner in both CEF cells and primary lens cells^18, 22^. Moreover, adhesion function by Cx50 does not require a connexin partner from neighboring cells. Cx50 mediates heterotypic adhesion and, intriguingly, the expression of other lens connexins in the partner cell is able to reduce the adhesive effect of Cx50. It is likely that the expression of other connexins on the cell surface may partially interfere with the interaction of the adhesion partner molecule(s) with Cx50. The adhesion partner(s) may not necessarily be a protein as the involvement of other molecules on the cell surface such as lipids or carbohydrates cannot be excluded. Additionally, we found that mutation of two positively charged amino acid residues at the E2 domain abolished adhesive function of Cx50, which implies the possible interaction between extracellular positively charged residues of Cx50 and negatively charged lipid bilayers. Further investigation in this area is warranted.

There are two extracellular loop domains (E1 and E2) of connexin molecules with the E1 domain involved in the formation of the gap junction channel and E2 in the docking compatibility in heterotypic gap junction channels (see review ref. 33). Deletion of either extracellular domain impairs protein stability and intracellular delivery of connexin molecules to the cell surface^34^. Here, we used a competition assay with the fusion proteins containing either the E1 or E2 domain in order to define the involvement of a specific extracellular domain. We found that only the E2 domain inhibited cell-cell adhesion whereas E1 had a minimal effect. The effect of the E2 domain is specific for Cx50 as this domain does not inhibit cell adhesion mediated by AQP0. We identified 5 amino acid residues by which mutating any one of them compromised the capability of E2 fusion protein in the inhibition of cell adhesion as well as lens cell differentiation. These five residues are conserved among connexin subtypes. A similar level of the inhibition of Cx50 adhesion was also observed with Cx43E2 fusion protein, which suggests that Cx43 E2 domain with those conserved residues like Cx50E2, is likely to disrupt these interactions. These data support the notion that several conserved residues of E2 domain are involved in the adhesion function of Cx50. It is likely that other unique residues on Cx50 might interact with these conserved residues to mediate the adhesive function. Four out five residues (except C190) are located in the protein motifs containing either α-helix or β-sheet; two are positively charged and one is involved in forming a hydrogen bond. Two residues that abolish the inhibitory effect of E2-GST are C185 and C190. According to the Cx26 hemichannel structure (formed by 6 monomers), cysteines in the 2nd extracellular loop domains are engaged in formation of disulfide bonds with cysteines in the 1st extracellular loop of the same monomer. The sequence comparison of E2 and 4^th^ transmembrane domains of Cx26 and Cx50 and structure predictions are shown in Fig. S3. Because there are 3 cysteines in E2, it is possible that in the E2-GST fusion protein C185 and C190 formed disulfide bonds with other cysteines in the same or a different E2 domain of Cx50. The cysteines are conserved residues in the E2 domain. It is possible that the cysteines and the critical residues identified in Cx50, may interact with other residue(s), not in the other two connexins, critical for forming certain unique structure to mediate cell-cell adhesion. The secondary structures of E2 domain and electrostatic interactions may play crucial roles in facilitating the adhesive function of Cx50 with either the partner Cx50 or possibly negatively charged phospholipids or other proteins from neighboring cells.

Cx50, unlike Cx46, the other connexin predominantly expressed in lens fibers, plays an important role in lens development and lens epithelial to fiber cell differentiation, and Cx50 deficiency results in a small lens^25–27, 35^. We also show in our earlier study that the C-terminus of Cx50 is involved in promoting lens cell differentiation. However, Cx50 C-terminus along is not sufficient for lens cell differentiation. Cell-cell adhesion is known to be crucial for the process of normal cell proliferation and differentiation. In this study, we show that cell-cell adhesion mediated by Cx50 is an important part in the process of lens cell differentiation. Primary chick lens cultures undergo an autonomous program of cell differentiation, in which monolayer epithelial cells gradually differentiate into lens fiber-like cells with the formation of lentoids structures and the expression of lens fiber cell markers including AQP0 and crystallin molecules^28, 36^. By taking advantage of this system, we found that the E2 domain, which disrupted the cell-cell adhesion by Cx50, compromised lens cell differentiation with the reduction of lentoid formation and expression of AQP0. Furthermore, we showed that identical group of amino acid residues on Cx50 E2 domain participate for both cell adhesion and lens cell differentiation, supporting the notion that cell adhesive function of Cx50 directly influences lens cell differentiation. Together, this study uncovers a unique mechanism for Cx50 in cell adhesion, which is likely to play a crucial role in lens cell differentiation and development.

## Materials and Methods

### Materials

Fertilized white leghorn chicken eggs were obtained from Texas A&M University, Department of Agriculture & Poultry Science (College station, TX) and incubated in a humidified chicken egg incubator. Rabbit anti-chick AQP0 polyclonal antibody was generated from rabbits against chicken AQP0 CT and affinity purified as previously described^37^. Anti-FLAG tag antibody (Cat#600-401-383) was obtained from Rockland Immunochemicals (Pottstown, PA). Paraformaldehyde (PFA, 16%) was from Electron Microscope Science (Fort Washington, PA). Dulbecco’s Modified Eagle Medium (DMEM), 0.25% Trypsin-EDTA solution, penicillin/streptomycin were purchased from Invitrogen (Carlsbad, CA, USA). Fetal bovine serum (FBS) was obtained from Hyclone Laboratories (Logan, UT, USA). All other chemicals were obtained from either Sigma-Aldrich (St. Louis, MO) or Fisher Scientific (Pittsburgh, PA).

### Cell Culture, Retroviral Expression, Preparation of Cell Membrane Extracts and Western Blotting

CEF cells were cultured in DMEM plus 10% fetal bovine serum and 2% chick serum. HEK293 cells were cultured in DMEM medium plus 10% fetal bovine serum. Cells were infected with retrovirus or lentivirus after the second day of culturing. After reaching confluence, cells were digested with 0.05% trypsin and passaged. Confluent cells were collected in ice-cold lysis buffer containing 1 mM PMSF, 1 mM NaVO_4_, and 0.1 mM leupeptin and lysed with a 26 gauge needle. Cell lysates were centrifuged for 5 min at 1000 g to remove cell debris. Crude membrane extracts were then prepared by centrifuging at 100,000 g for 30 min (TLA55 rotor, Beckman Coulter, Brea, CA, USA) at 4 °C and pellets were resuspended in the lysis buffer, pH 7.4. Crude cell membrane extracts were boiled in 0.6% SDS and separated on a 10% SDS-PAGE gel. We loaded equal amount of total proteins in each lane of SDS-PAGE. The equal protein loading was achieved through the measurement of protein concentration using microBCA assay (Pierce, Rockford, IL, USA). Western blotting was performed by probing with anti-FLAG (1:1000 dilution) or anti-β-actin (1:5000 dilution) antibody. Primary antibodies were detected with goat anti-rabbit IgG conjugated IRDye® 800CW and goat anti-mouse IgG conjugated IRDye® 680RD (1:15000 dilution) using a Licor Odyssey Infrared Imager (Lincoln, NE, USA). The intensity of the bands on western blots was quantified.

### Preparation of High-Titer Recombinant Retroviruses and Lentiviruses

CEF cells were prepared as previous described^38^ and tested for possible contamination with mycoplasma. Recombinant retroviral DNA constructs and high titer retroviruses containing Cx43, Cx46, Cx50 and AQP0 were prepared based on our protocol described previously^5, 38^. Briefly, high-titer recombinant retroviruses were generated through transfection of these DNA constructs into CEF cells (1–5 × 10^8^ cells per ml). 4.5 µg DNA constructs containing connexins or AQP0 were transfected into 60 mm CEF cell at 50–70% confluency using lipofectamine according to the manufacturer’s instructions (Thermo Fisher Scientific). Crude membrane extracts of transfected CEF cells were prepared and immunoblotted with rabbit anti-FLAG antibody to examine the expression of connexins or AQP0. Conditioned media was collected and concentrated to make high titer retrovirus.

For preparation of mouse Cx50 lentiviral construct, mouse Cx50 cDNA was cloned into a lentivirus transfer vector (pSDM-GFP), which was packed using pMD2.G (Addgene) and psPAX2 (Addgene) by co-transfection using lipofectamine according to the manufacturer’s instructions (Thermo Fisher Scientific) in HEK293 cells. Transfected HEK293 cells were cultured for two weeks to reach almost 100% viral expression, and then collected for use.

### Protein Pull down assay

The sequences of Cx50 E1 and E2 domains were determined using the most acceptable online analysis tools on both protein transmembrane domain prediction (https://www.ncbi.nlm.nih.gov/protein/NP_990328.1), and transmembrane domain and membrane topology prediction (TMHMM Server, v. 2.0 -CBS -DTU http://www.cbs.dtu.dk/services/TMHMM/; http://embnet.vital-it.ch/software/TMPRED_form.html) on chicken Cx50. The GST fusion protein containing E1 or E2 was prepared with PCR, cloning and isolation with glutathione-conjugated beads. Purified fusion protein E1-GST, E2-GST or GST as control was first preincubated with glutathione–agarose beads at 4 °C for 2 hrs and the beads were washed three times with ice-cold lysis buffer (20 mM Tris-HCl, 200 mM NaCl, 1 mM EDTA, 0.5% NP40, pH 8.0) plus protease inhibitors to eliminate non-binding GST-fusion protein to the beads. Crude membrane extracts obtained from CEF cells expressing exogenous Cx50, Cx50 or RCAS(A) vehicle were incubated with the corresponding E1-GST, E2-GST or GST bound glutathione-agarose beads overnight at 4 °C. The beads were then washed four times with ice-cold GST lysis buffer plus protease inhibitors. The pull-down samples were isolated from the beads by boiling in SDS sample loading buffer for 5 min and subjected to SDS-PAGE and immunoblotting.

### Cell-Cell Adhesion Assay

The cell adhesion assay was performed based on a modified protocol^12^ with some modifications. Briefly, CEF cells infected with RCAS(A) retrovirus as a vehicle control, or recombinant RCAS(A) containing connexins or AQP0 were grown to confluence. HEK293 cells transfected with pSDM-GFP as a vehicle control, or pSDM-mCx50 were grown to confluence. Equal numbers of donor cells preloaded with DiI dye were parachuted over the unlabeled recipient cells at a 1:50 donor to receiver ratio. For CEF cells, equal numbers of donor cells preloaded with DiI dye were premixed with GST, Cx50E1-GST, Cx50E2-GST or Cx43E2-GST fusion protein at 100 ng/ml for 30 min at room temperature and parachuted over the unlabeled recipient cells at a 1:50 donor to receiver ratio. After incubation for 1.5 hrs at 37 °C, cells were washed with PBS with Ca^2+^ and Mg^2+^ to remove non-adherent cells and examined under an Olympus fluorescence microscope. At least five representative images for each condition tested were used to assess cell adhesion per measurement.

### Lens tissue paraffin sections and hematoxylin and eosin (H&E) staining

All animals were housed and studied in accordance with NIH Animal Care and Use Committees (ACUC) guidelines and the animal protocols approved by the University of Texas Health Science Center Institutional Animal Care and Use (IACUC) Committee. Day-15 mice of WT or Cx50 KO mice were euthanized, and eyeballs were isolated and fixed in 2% PFA at 4 °C for overnight, dehydrated with ethanol and xylene, embedded in paraffin and sectioned 4–5 µm in thickness. The tissue sections were mounted to glass slides, stained with hematoxylin & eosin and observed under an Olympus IX70 microscope. Images were recorded under 20X magnification using an Olympus camera.

### Thin-section Electron Microscopy

Freshly isolated 14-day old mouse lenses were fixed in an improved fixative containing 2.5% glutaraldehyde, 0.1 M cacodylate buffer (pH 7.3), 50 mM L-lysine and 1% tannic acid for 2 hr at room temperature as previously described^39^. Each lens was then mounted on a specimen holder with superglue and cut into 200 µm slices with a Vibratome. Each lens was carefully oriented on the specimen holder such that either a cross or longitudinal section of cortical fibers could be obtained initially with a Vibratome. Lens slices were then post-fixed in 1% aqueous OsO_4_ for 1 hr at room temperature, rinsed in dH_2_O and stained en bloc with 0.5% uranyl acetate in 0.15 M NaCl overnight at 4 °C. Tissue slices were dehydrated through graded ethanol and propylene oxide, and embedded in Polybed 812 resin (Polysciences, PA, USA). Thick sections (1 µm) cut with a diamond knife were stained with 1% toluidine blue and examined with a light microscope to select the area of interest. Thin sections (80 nm) were cut with a diamond knife, stained with 5% uranyl acetate followed by Reynold’s lead citrate and examined in a JEOL 1200EX electron microscope.

### Lens Primary Culture and Immunostaining

Primary lens cell cultures were prepared by a modified method described previously^28^. Lenses from 11-day-old chick embryos were dissected, washed with TD buffer (140 mM NaCl, 5 mM KCl, 0.7 mM Na_2_HPO_4_, 5 mM glucose and 25 mM Tris at pH 7.4), and digested with 0.1% trypsin in TD buffer at 37 °C, and then broken into individual cells in M199 media plus 10% FBS. Cells were collected and resuspended in M199 media. Living cells were then counted and seeded at 4 × 10^5^ cells per well of 12-well culture plates. At the 2nd day of cell culturing, GST or GST-E1 GST-E2 fusion protein (diluted in M199 to 100 ng/ml) were added to primary lens cultures. The cultures were incubated at 37 °C with 5% CO_2_ and media was changed every day. In the beginning of culturing, only monolayer lens epithelial cells proliferated on the culture plates, but not fiber cells. After 4–5 days, lens epithelial cells became confluent and began to differentiate and form fiber-like lentoid structures. After 14 days of primary culture when the number of lentoids plateaued, primary culture cells were fixed in 2% PFA at room temperature for 30 min, blocked with blocking solution containing 2% goat serum, 1% BSA, 2% fish gelatin and 0.25% Triton X-100 in PBS buffer. Expression of AQP0 in lentoids was examined with rabbit anti-AQP0 antibody (1:30 dilution). Primary antibody was detected by goat anti-rabbit conjugated with Alexa Fluor 488 and examined under fluorescence microscope for cell adhesion. At least eight representative images for each condition tested were used to measure the AQP0 expression level and the data were quantified from at least three independent experiments. Expression level was quantified with Image J software (NIH).

### Preparation Of GST-Cx50(E1) And Cx50(E2) Fusion Proteins, Generation Of Site Mutants and Determination of Half-lives

A GST-tagged fusion protein containing the E1 or E2 domain of Cx50 was prepared as followed. Briefly, a cDNA fragment encoding the E1 or E2 domain of Cx50 was generated by PCR using a chick Cx50 cDNA clone as a template with the following pair of primers: E1: sense, 5′-CACAGGATCCGTATGGGGAGATGAACAGT-3′; antisense, 5′-CTTCGAATTCTCAGTGGGAGATGGGGAAGGCCTCA-3′; E2: sense, 5′-CACAGGATCCGGCTTCCGCATTCTCCCCCTTTACCG-3′; antisense: 5′-CTTCGAATTCTCAGATGGTCTTCTCTGTGG-3′, and were subcloned into the expression vector pGEX-2T. The sequences were confirmed at the UTHSCSA DNA core facility. The recombinant fusion protein was expressed in DH5α *E. coli*, induced with 0.5 mM isopropyl β-D-1-thiogalactopyranoside (IPTG) and then isolated and purified with glutathione-conjugated agarose beads.

Cx50 single site mutants were generated with the QuikChange^TM^ site-directed mutagenesis kit according to the manufacturer’s instruction with the primers shown in Table 3. PCR primers were synthesized and constructs were sequenced at the University of Texas Health Science Center at San Antonio (UTHSCSA) DNA Core Facility.

**Table 3:** The primers used for site-directed mutagenesis studies.

Mutants	Sense primers (5′-3′)	Antisense primer (5′-3′)
C185A	CCCCTTTACCGCGCGGGGCGGTGGCCCTG	CAGGGCCACCGCCCCGCGCGGTAAAGGGG
C190A	TGTGGGCGGTGGCCCGCGCCCAACCTAGTGGAC	GTCCACTAGGTTGGGCGCGGGCCACCGCCCACA
C196A	CCAACCTAGTGGACGCGTTTGTCTCCAGG	CCTGGAGACAAACGCGTCCACTAGGTTGG
R200A	GACTGTTTTGTCTCCGCGCCCACAGAGAAGACC	GGTCTTCTCTGTGGGCGCGGAGACAAAACAGTC
P201A	GTTTTGTCTCCAGGGCGACAGAGAAGACC	GGTCTTCTCTGTCGCCCTGGAGACAAAAC
T202A	GTTTTGTCTCCAGGCCCGCGGAGAAGACCATCTGAG	CTCAGATGGTCTTCTCCGCGGGCCTGGAGACAAAAC
E203A	GTTTTGTCTCCAGGCCCACAGCGAAGACCATCTGAG	CTCAGATGGTCTTCGCTGTGGGCCTGGAGACAAAAC
K204A	CTCCAGGCCCACAGAGGCGACCATCTGAGAATTC	GAATTCTCAGATGGTCGCCTCTGTGGGCCTGGAG

Confluent cultures of CEF cells infected with RCAS-Cx50 lentivirus were incubated with 100 ng/ml of GST-Cx50E1 or GST-Cx50E2 fusion protein for various time periods and cell culture media were collected at the same time. The supernatant were centrifuged at 7000 g for 5 min in order to remove cells and debris. Equal volume of samples was analyzed on 15% SDS-PAGE gels and Western blot analysis. Nitrocellulose membranes were incubated with rabbit anti-GST antibody (1:1000 dilution) and followed by corresponding secondary antibody. The pixel densities of reactive bands were quantified using Image J. The half-lives of GST-fusion proteins were calculated as one phase decay because of one parameter (reduction of GST reactivity) using the following formula: T_1/2_ = ln(2)/k with (−k) representing the slope of the curves obtained from different time periods as indicated: k = ((C1 − C2)/(t2 − t1))/Ci with C1 = Concentration at time t1, C2 = Concentration at time t2 and Ci = Concentration at initial time.

### Statistical Analysis

All data were analyzed with GraphPad Prism 5 Software (GraphPad Software, La Jolla, CA). Two group comparisons were performed using a paired design t-test and multiple comparisons were done with one-way ANOVA and Newman-Keuls multiple comparison test. The data were presented as the mean ± SEM of at least three independent experiments. Statistical significance was designated for analyses with P < 0.05. Asterisks in all figures indicate the degree of significant differences compared to controls, *P < 0.05; **P < 0.01; ***P < 0.001.

## Electronic supplementary material

Supplementary information
