# Supplementary material for: Connexin 50 Functions as an Adhesive Molecule and Promotes Lens Cell Differentiation
Source: Sci Rep. 2017 Jul 13;7:5298. doi: 10.1038/s41598-017-05647-9 (PMC5509658; doi:10.1038/s41598-017-05647-9)

Connexin 50 Functions as an Adhesive Molecule and Promotes Lens Cell Differentiation

Zhengping Hu[1,2], Wen Shi[1,2], Manuel A. Riquelme[1], Qian Shi[3], Sondip Biswas[4], Woo-Kuen Lo[4], Thomas W. White[5], Sumin Gu[1] and Jean X. Jiang[1*]

Department of [1]Biochemistry and Structural Biology and [3]Physiology, University of Texas Health Science Center, San Antonio, TX;

[2] The second Xiangya Hospital, Central South University, Changsha, China;

[4]Department of Neurobiology, Morehouse School of Medicine, Atlanta, GA;

[5]Department of Physiology and Biophysics, Stony Brook University, Stony Brook, NY

To whom correspondence should be addressed: Dr. Jean X. Jiang, Department of Biochemistry and Structural Biology, University of Texas Health Science Center, 7703 Floyd Curl Drive, San Antonio, TX 78229-3900, Telephone: (210) 562-4094; E-mail: jiangj@uthscsa.edu

Supplemental Figure Legends:

Figure S1. Cx50 expressing CEF cells do not form functional gap junction channels with parental CEF cells. CEF cells were infected with recombinant retrovirus containing Cx50. The donor CEF cells (indicated by *) with or without Cx50 expression were loaded with calcein-AM (green fluorescence cells) for 45 min and "parachuted" on top of confluent receipt CEF cells with or without Cx50 and incubated for 90 min. The phase and fluorescence images were taken by an Olympus microscope.

Figure S2. Surface expression of P88S in CEF cells. CEF cells were infected with recombinant RCAS(A) containing Cx50, Cx50P88S or RCAS(A) vehicle. The cells were labeled with wheat germ agglutinin (WGA) (red) and immunostained with anti-Cx50 antibody (green). The white arrows indicate the staining of Cx50 and P88S mutant on the cell surface.

Figure S3. Sequence comparison of E2 and 4th transmembrane domain of Cx50 with those of Cx26 and structure prediction based on Cx26 X-ray structure.

Figure S4. Determination of Cx50E1-GST and Cx50E2-GST stability. Cx50E1 or Cx50E2 GST fusion protein was incubated with CEF cells infected with RCAS(A)-Cx50. The cell media were collected at various time periods. The amount of fusion proteins was determined by western blotting with anti-GST antibody and band intensity of fusion proteins was quantified and graphed. The half-lives of fusion protein were determined as described in the Materials and Methods.

Figure S5. Inhibition of cell adhesion by Cx43E2 fusion protein. CEF cells were injected with RCAS(A) vehicle (control) or RCAS(A) containing Cx50 and incubated with GST-fusion protein containing E2 domain of Cx43 or Cx50. Cell adhesion assay was conducted and adherent cells were quantified and compared with the vehicle control. The data are presented as the mean ± SEM. n = 3. **, $P < 0.01$.

Fig. S1

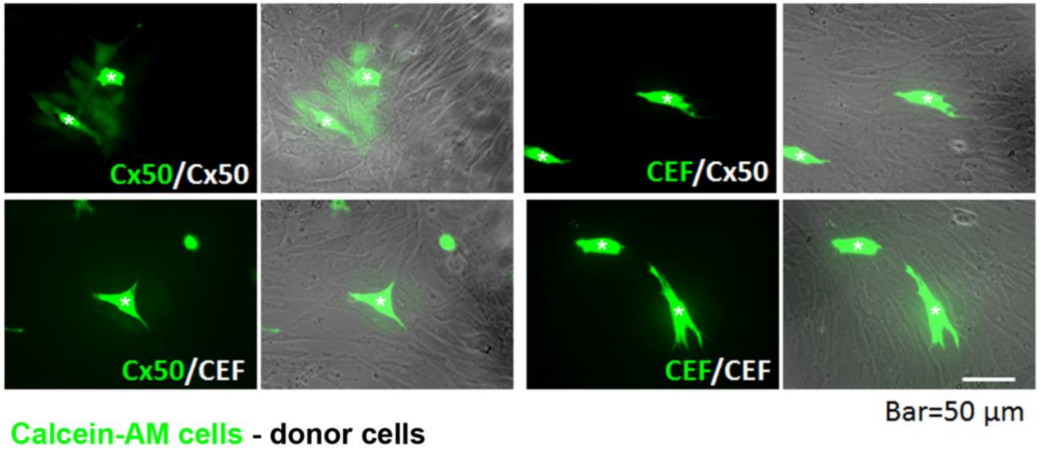
Cx50/Cx50
CEF/Cx50
Cx50/CEF
CEF/CEF
Bar=50 µm
Calcein-AM cells - donor cells

Fig. S2

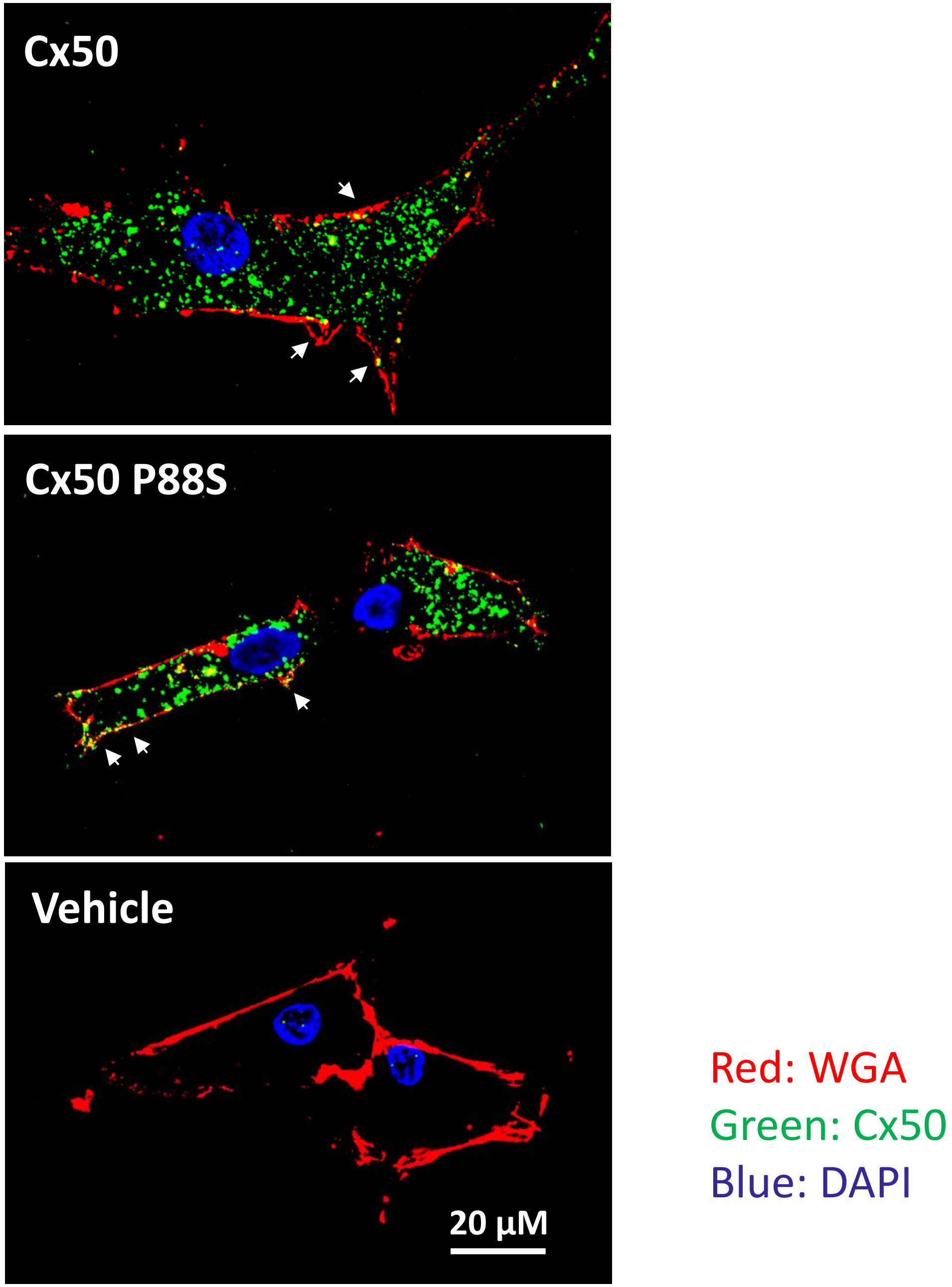

Fig. S3

```
                           E2                          TM4
chCx50     176 GFRILPLYRCGRWPCPNLVDCFVSRPTEKTIFIMFMLVVAAVSLFLNLVEISHLI 230
                ||.||++|.|+++||||+||||||||||||||++||+++|++|+++|++|+.|++
Cx26       160 GFSMQRLVKCNAWPCPNTVDCFVSRPTEKTVFTVFMIAVSGICILLNVTELSYLL 191
ss_pred        SSCCCSEEEECCTTSSSCEEEECSSHHHHHHHHHHHHHHHHHHHHHHHHHHHHHH
```

Symbols between alignment represent AA match: ‘|’ very good; ‘+’ good; ‘·’ neutral.
Symbols in “ss_pred” (structure prediction) represent: S: Bend; C: Random coil; T: Hydrogen bond turn; E: β-sheet; H: α-helix

Fig. S4

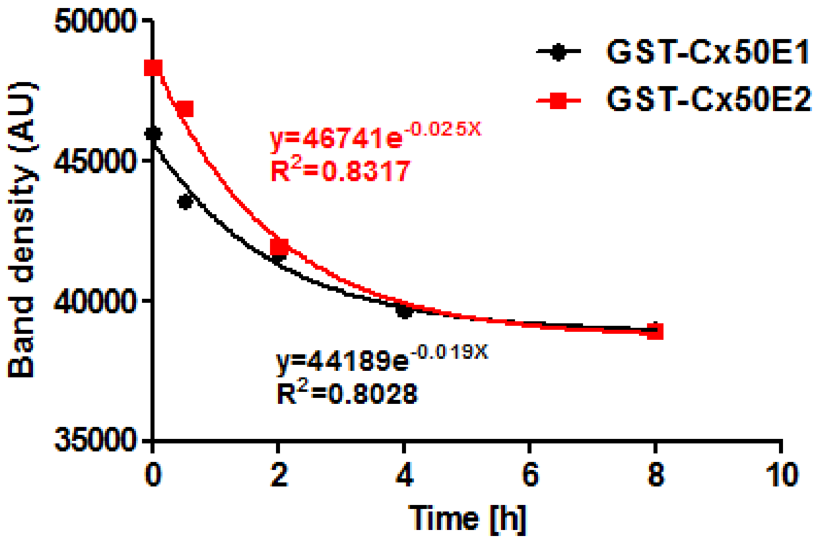

Band density (AU)
50000
45000
40000
35000
0
2
4
6
8
10
Time [h]
GST-Cx50E1
GST-Cx50E2
y=46741e-0.025X
R2=0.8317
y=44189e-0.019X
R2=0.8028

Fig. S5

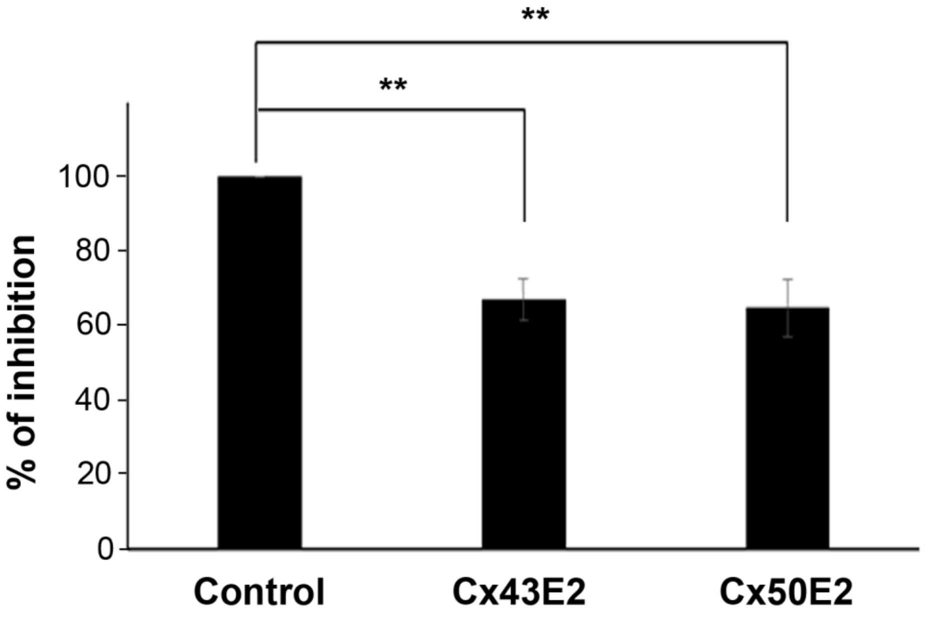

Supplement: Supplementary file 1 — Supplementary information [file 41598_2017_5647_MOESM1_ESM.pdf]
